# IL-1α is a DNA damage sensor linking genotoxic stress signaling to sterile inflammation and innate immunity

**DOI:** 10.1038/srep14756

**Published:** 2015-10-06

**Authors:** Cohen Idan, Rider Peleg, Voronov Elena, Tomas Martin, Tudor Cicerone, Wegner Mareike, Brondani Lydia, Freudenberg Marina, Mittler Gerhard, Ferrando-May Elisa, Charles A. Dinarello, Apte N. Ron, Schneider Robert

**Affiliations:** 1 Institut de Génétique et de Biologie Moléculaire et Cellulaire, CNRS UMR 7104, INSERM U 964, Université de Strasbourg, 67404 Illkirch, France; 2 Max Planck Institute of Immunobiology and Epigenetics, 79108 Freiburg, Germany; 3 The Shraga Segal Department of Microbiology, Immunology and Genetics, Faculty of Health Sciences, Ben-Gurion University of the Negev, 84105 Beer-Sheva, Israel; 4 Bioimaging Center and Center for Applied Photonics, Department of Biology and Department of Physics, University of Konstanz, Konstanz, Germany; 5 Department of Dermatology, Medical Center and Faculty of Biology, University of Freiburg, 79104 Freiburg, Germany; 6 Department of Pneumology, Faculty of Medicine, University of Freiburg, Freiburg, Germany; 7 BIOSS, Faculty of Biology III, University of Freiburg, Freiburg, Germany; 8 University of Colorado Denver, Aurora, CO USA

## Abstract

Environmental signals can be translated into chromatin changes, which alter gene expression. Here we report a novel concept that cells can signal chromatin damage from the nucleus back to the surrounding tissue through the cytokine interleukin-1alpha (IL-1α). Thus, in addition to its role as a danger signal, which occurs when the cytokine is passively released by cell necrosis, IL-1α could directly sense DNA damage and act as signal for genotoxic stress without loss of cell integrity. Here we demonstrate localization of the cytokine to DNA-damage sites and its subsequent secretion. Interestingly, its nucleo-cytosolic shuttling after DNA damage sensing is regulated by histone deacetylases (HDAC) and IL-1α acetylation. To demonstrate the physiological significance of this newly discovered mechanism, we used IL-1α knockout mice and show that IL-1α signaling after UV skin irradiation and DNA damage is important for triggering a sterile inflammatory cascade *in vivo* that contributes to efficient tissue repair and wound healing.

The cytokine interleukin-1 (IL-1) is a major mediator of an increasing number of systemic and local inflammatory diseases, but also functions to promote tissue repair and host defense against infection^1^. The IL-1 family consists of 11 members of which IL-1*α* and IL-1β have similar properties upon binding to the cell surface IL-1 receptor, however they display substantial differences related to their role in disease, tissue repair and immune defense^1^. Whereas both proteins are synthesized as precursors, the precursor form of IL-1α (precIL-1α) is, unlike the IL-1β precursor, highly active and is present in a broad range of healthy cells, especially in skin keratinocytes, epithelial cells of mucosal membranes and also endothelial cells^1^. IL-1α can serve as an initiator of the inflammatory process and mediates the initial neutrophil response at the site of inflammation^2^, before IL-1β exerts its role as pro-inflammatory cytokine. IL-1α intracellular levels are upregulated during several stress responses such as hypoxia^3^, heat shock^4^ or UV exposure. When cells undergo necrotic cell death, precIL-1α is immediately released and initiates a pro-inflammatory “alarm signal” to the nearby cells.

Due to a nuclear localization signal (NLS), the precursor form of IL-1*α* is also found in the nucleus and can act as a dual function cytokine having an intracellular as well as extracellular mechanism of action^5^, similar to HMGB1, IL-33 and IL-37^6^. Previous studies demonstrated that IL-1*α* interacts with the histone acetyltransferases p300/pCAF and shows transcription activation capabilities that depend on its N-terminus^7^,^8^.

Despite the fact that IL-1α is a prominent “alarmin” released during necrosis, evidence also suggests that IL-1α may be secreted by cells that maintain their integrity^9^. For example, heat-shock^4^ and various genotoxic stresses such as UV radiation^10^, DNA cross-linking^11^, oxidative stress^12^ and persistent DNA damage^13^, promote IL-1α secretion. In addition, extensive oxidative DNA damage occurs in a number of diseases such as Systemic Sclerosis (Ssc) and Rheumatoid Arthritis (RA)^13^,^14^,^15^ with accompanying levels of IL-1α in patients’ serum^16^,^17^. Altogether, these reports suggest that cells that have been subjected to non-lethal stress can actively secrete the precursor form of IL-1α in addition to its passive release by necrotic cells. However, up to now, no signaling mechanism or known secretion pathways were shown to link those events.

## Results and Discussion

**Figure 1:**
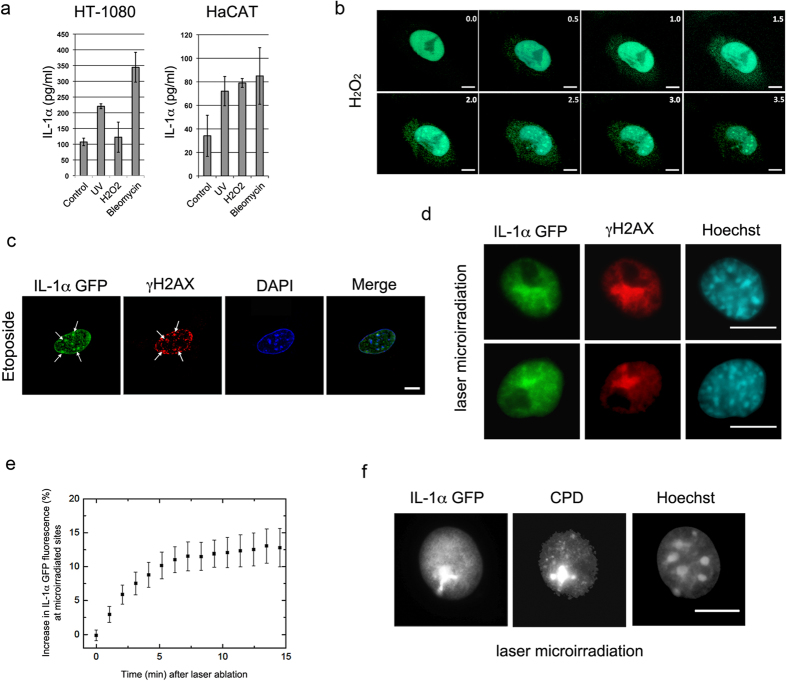
IL-1α is recruited to DNA damage sites and secreted after genotoxic stress. (**a**) Human HT1080 fibrosarcoma and HaCaT human keratinocytes were subjected to genotoxic stresses: UV irradiation (5 mJ/cm^2^), 10 mM H_2_O_2_ or 50 μgr/ml Bleomycin. 16 h post exposure, hIL-1α ELISA was used to measure secreted IL-1α in cell supernatants. All experiments were performed in triplicates and data are expressed as mean ± SD. (**b**) Nuclear/cytoplasmic re-localization of IL-1α after DNA damage. Live cell imaging of B16 melanoma cells expressing GFP-IL-1α during treatment with 100 *μ*M H_2_O_2_. Images were collected every 30 min for a period of 24 h. Representative images from indicated time points are shown (for full video see Supplementary Video 1, for averaged fluorescence intensities see also Supplementary Figure 1b) White scale bars, 20 *μ*m. (**c**,**d**) Nuclear IL-1α co-localizes with γH2AX foci after genotoxic stress. (**c**) B16 melanoma cells expressing GFP-IL-1α were treated with Etoposide 10 µg/mL for 2 h or (**d**) microirradiated with femtosecond laser pulses at λ = 775 nm (see also Supplementary Videos 2 and 3). After fixation of cells, detection of GFP-IL-1α, DAPI or immunostaining of γH2AX was preformed and visualized by confocal microscopy. White scale bars, 20 *μ*m (**e**) Recruitment kinetics of IL-1α to DNA damage sites. B16 melanoma cells expressing IL-1α–GFP were laser-microirradiated along a single line to induce DNA damage. Fluorescence intensity in the damaged region was measured up to 15 min from irradiaton in 1 min intervals. Data is expressed as mean ± SEM of increase in fluorescence intensity (n = 10 cells). (**f**) IL-1α localizes to Cyclobutane Pyrimidine Dimers (CPD) induced via laser microirradiation. B16 melanoma cells expressing GFP-IL-1α were laser irradiated and CPDs were visualized by immunostaining using specific antibodies. White scale bars, 20 *μ*m.

Since it had been reported that exposure to environmental factors that cause DNA damage may trigger precIL-1α secretion^4^,^10^ and affect the progression and severity of inflammatory diseases^12^,^14^,^15^,^16^,^18^,^19^, we examined the possibility that IL-1α could transduce signals from the nucleus to communicate chromatin damage to the surrounding tissue. We first confirmed that DNA damage indeed induces the secretion of IL-1*α* precursor. We used human keratinocytes and fibroblasts containing basal levels of the precIL-1α (Supplementary Figure 1a) to monitor the secretion of IL-1α after exposure to different genotoxic agents. As previously reported^11^,^13^, we detected increased levels of IL-1α in cell supernatants after exposure to various DNA damaging agents including H_2_O_2_, UV or Bleomycin (Fig. 1a). To gain insight into the intracellular dynamics of IL-1α upon DNA damage, we next used an IL-1α–EGFP expressing cell line (previously used in^5^) and performed time-lapse analysis upon H_2_O_2_ treatment. Within 1.5 hours after exposure of cells to sub-lethal doses of H_2_O_2_ that generate large scale DNA damage, we observed a pronounced localization of IL-1α to nuclear foci and an increase in the cytoplasmic IL-1α signal (n = 10) (Fig. 1b, Supplementary Video 1 and Supplementary Figure 1b). We next treated cells with etoposide, a drug that prevents re-ligation of the DNA strands^20^ and causes DNA strands breaks. Interestingly, we found that IL-1α can co-localize with phosphorylated H2A.X (γH2AX) foci that mark DNA damage sites (Fig. 1c and Supplementary Figure 1c). When DNA was damaged by laser microirradiation, IL-1α also co-localized with γH2AX foci and became enriched at these DNA lesions within 5 to 10 minutes (Fig. 1d,e Supplementary Videos 2 and 3). Furthermore, we observed localization of IL-1α at laser-induced Cyclobutane Pyrimidine Dimers (CPD), also induced by these laser pulses at λ = 775 nm (Fig. 1f).

The IL-1α foci after genotoxic stress induction by low UV irradiation (Supplementary Figure 2a **lower panel)** were distinct from the broad immobile bulk IL-1α foci that we previously characterized during apoptosis (Supplementary Figure 2a **upper panel)** and they are largely excluded from PML bodies (Supplementary Fig. 2b)^5^. Together, this data shows that IL-1α can localize to DNA damage sites and can be secreted without the loss of cellular integrity.

**Figure 2:**
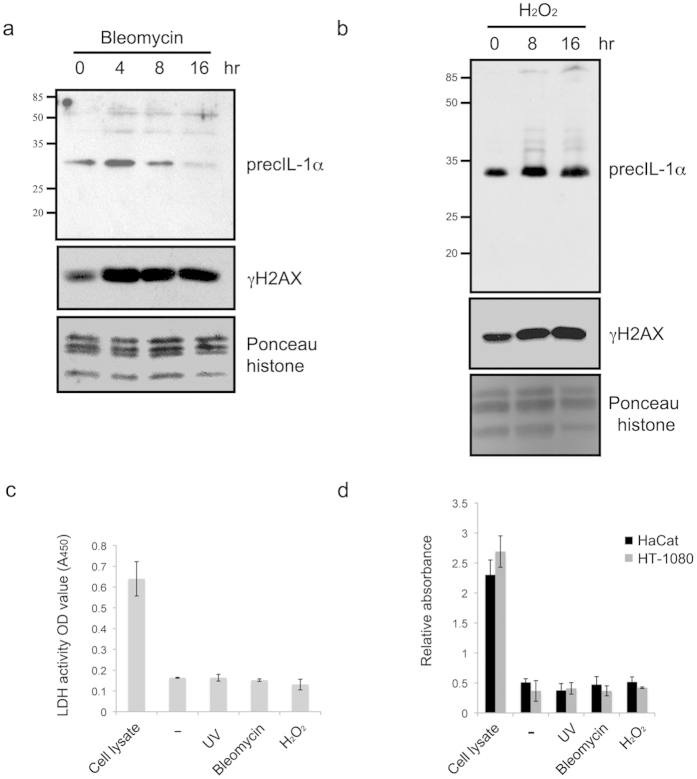
IL-1α is released after genotoxic stress in the absence of detectable processing or cell integrity breakdown. Total cell lysates from human HT1080 fibrosarcoma exposed to (**a**) bleomicin or (**b**) H_2_O_2_ were assessed for processing of precIL-1α. For this total proteins were extracted from cell pellets at indicated time points and separated over 15% SDS PAGE. IL-1α or γH2AX western blots were preformed. Histones were used as loading control. (**c**) Detection of necrosis by release of lactate dehydrogenase activity. HT1080 fibrosarcoma were exposed to several genotoxic stresses as above (UV, H_2_O_2_ and bleomycin). Supernatants were collected and analyzed for release of lactate dehydrogenase. Data is expressed as mean ± SD of 3 independent experiments. (**d**) Determination of mono- and oligonucleosomes released by necrotic cell death in treated (as in Fig. 2c) HT1080 fibrosarcoma or HaCaT keratinocytes. Data is expressed as mean relative absorbance (A_405 nm_−A_490 nm_) ± SD of 3 independent experiments.

IL-1α can be cleaved by the Ca^2+^ dependent protease calpain or several other proteases, such as CTL/NK-granzyme-B, mast cell chymase or neutrophil elastase, that may increase its biological activity (providing more adjuvant activity *in-vivo*, or activation of cells *in-vitro*)^21^. This cleavage is not required for its release from stressed or dying cells or for binding to Interleukin Receptor-1 IL-1R1^1^. Processing of IL-1α precursor allows separation of the NLS containing domain from the receptor activating mature part of the molecule; therefore, retention of the protein in the nucleus is prevented. Although cellular stresses can effect the levels of IL-1α transcript, it is not clear if they also influence IL-1α processing to the mature form which is secreted via the poorly understood, ER/Golgi-independent unconventional protein secretion pathway^1^. Upon the cellular genotoxic stresses conditions used, we could not observe obvious precIL-1α processing (Fig. 2a,b) and also not substantial cell necrosis (Fig. 2c,d) or major apoptosis (Supplementary Fig. 2c).

**Figure 3:**
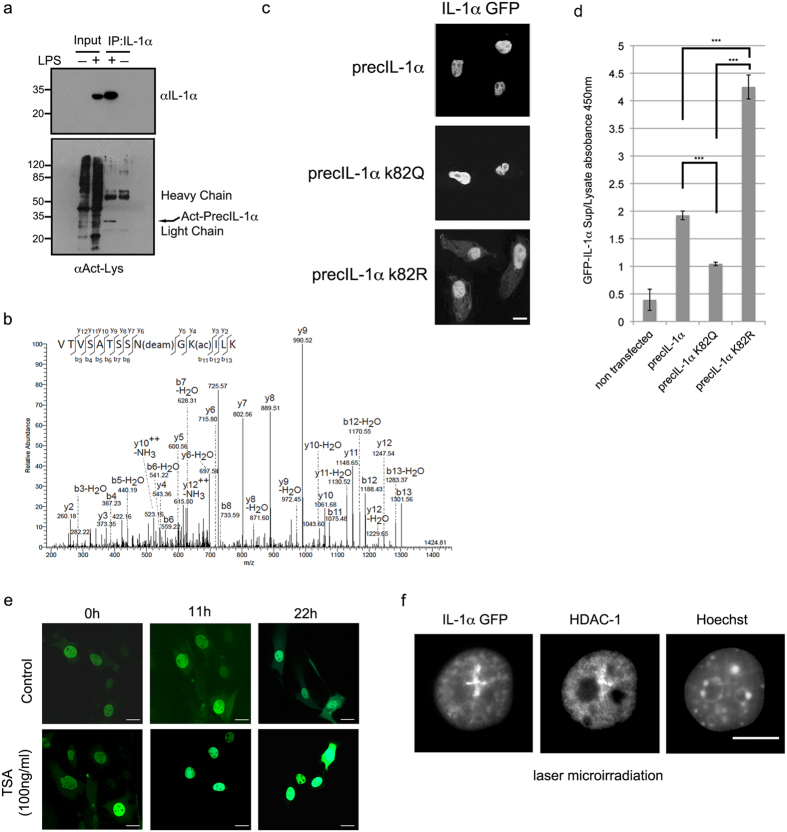
IL-1α acetylation within the nuclear localization sequence impacts on IL1α subcellular localisation. (**a**) IL-1α precursor is recognized by a pan acetyl antibody. Endogenous IL-1α was immunoprecipitated (IP) from nuclear extracts of Raw 264.7 cells, either induced or non-induced with 100 ng/ml LPS. Total IP proteins were separated over 15% SDS PAGE, transferred to nitrocellulose membranes and blotted with anti-mouse IL-1α (top panel) or anti-Kac (bottom panel). Acetylated IL-1α is marked by arrows and IP antibody light and heavy chain signals are indicated. (**b**) Annotated MS/MS spectrum of the tryptic peptide VTVSATSSN(Deam)GK(Acetyl)ILK (MH2 + 724.40 Da) showing acetylation of IL-1α (Uniprot ID P01582) at K82 and N80 deamidation. (**c**) PrecIL-1α K82 mutants affect IL-1α sub-cellular localization. Confocal microscopic analysis of GFP tagged WT IL-1α and mutations of precIL-1α K82 to glutamine (precIL-1α K82Q, mimicking acetylation) and to arginine (precIL-1α K82R non-acetylateable). White scale bars, 20 *μ*m (**d**) IL-1α K82 mutations reduce cytokine secretion after DNA damage. Mouse B16 cells were transfected with the indicated GFP IL-1α plasmids. The cells were then subjected to 100 μM H_2_O_2._ 16h after stress induction levels of secreted GFP IL-1α in cell growth medium was measured using a GFP ELISA. GFP IL-1α levels in cell lysates were used to normalize for transfection efficiencies and non-transfected cells were used as negative controls. Data are expressed as mean ± SD of three independent experiments. (**e**) Histone deacetylase inhibition by TSA increases IL-1α nuclear localization. Images of cells expressing GFP IL-1α either non-treated (control) or treated with TSA (100 ng/ml) were collected every hour for 22 h and representative images for three time points (0, 11 and 22 hours) are shown (For averaged fluorescence intensities of nuclear/cytoplasmic ratios see Supplementary Figure 1b). (**f**) HDAC-1 and IL-1α can co-localize at DNA damage lesions. Cells expressing GFP IL-1α were laser-microirradiated for the induction of DNA damage. Localization of HDAC-1 and IL-1α–GFP were visualized by confocal microscopic analysis.

Since the function and dynamics of many DNA damage response factors and complexes involved in DNA repair are regulated by posttranslational modifications (PTM), we examined the possibility that the cytoplasmic localization of IL-1α after DNA damage is also regulated by PTMs. To investigate the role of PTMs in IL-1α function we searched for novel IL-1α modifications. By using immunoprecipitation (IP), we observed that mouse IL-1α protein is strongly recognized by an anti-Acetyl lysine (KAct) antibody (Fig. 3a), suggesting a novel IL-1α acetylation. To corroborate this finding and to map the acetylation sites we performed mass spectrometry analysis and detected acetylation of lysine K82 within the IL-1α NLS (Fig. 3b). To better understand the role of IL-1α acetylation, we expressed GFP tagged IL-1α mutated on K82 to an arginine (K82R) or glutamine (K82Q), which are non-acetylatable or an acetylation mimic, respectively. Interestingly, expressing the K82Q mutation we detected increased IL-1α nuclear signal, whereas the K82R mutant seems to increase the cytoplasmic IL-1α signal without affecting overall cellular expression levels (Fig. 3c and Supplementary Figure 3a). Furthermore, the K82R mutation resulted in increased IL-1α release from UV irradiated cells, whereas release of the cytokine was lower when IL-1α was mutated to K82Q (Fig. 3d). Together these results strongly suggest that IL-1α acetylation can regulate its cellular localization.

Histone deacetylase inhibitors (HDACi) are widely used as anti-inflammatory agents^22^ and were shown to suppress IL-1α production from human peripheral blood mononuclear cells^23^. We therefore examined the effects of the HDACi Trichostatin A (TSA) on the localization of IL-1α in non-damaged cells. We found that TSA treatment increased the IL-1α nuclear signal and decreased the cytoplasmic signal over time, suggesting that HDACs could play a role in IL-1α sub-cellular localization (Fig. 3e and Supplementary Figure 3b). In addition, histone deacetylase inhibition by TSA reduced the secretion of the IL-1α precursor and of IL-6 from lipopolysaccharide (LPS) activated Raw 264.7 macrophages, showing that HDACs may also play a role as regulators of cytokine secretion (Supplementary Fig. 3c). Furthermore, the kinetics of IL-1α (Fig. 1e) recruitment to DNA damage sites resembles the recruitment of HDAC1 and HDAC2^24^, where they can deacetylate histone H3K56 and promote DNA double strand break (DSB) repair. Indeed, using IF we found a co-localization of HDAC-1 and IL-1α at DNA damage sites (Fig. 3f) suggesting that HDAC’s could indeed regulate IL-1α acetylation in response to DNA lesions and could be implicated in its nuclear/cytoplasmic re-localization.

**Figure 4:**
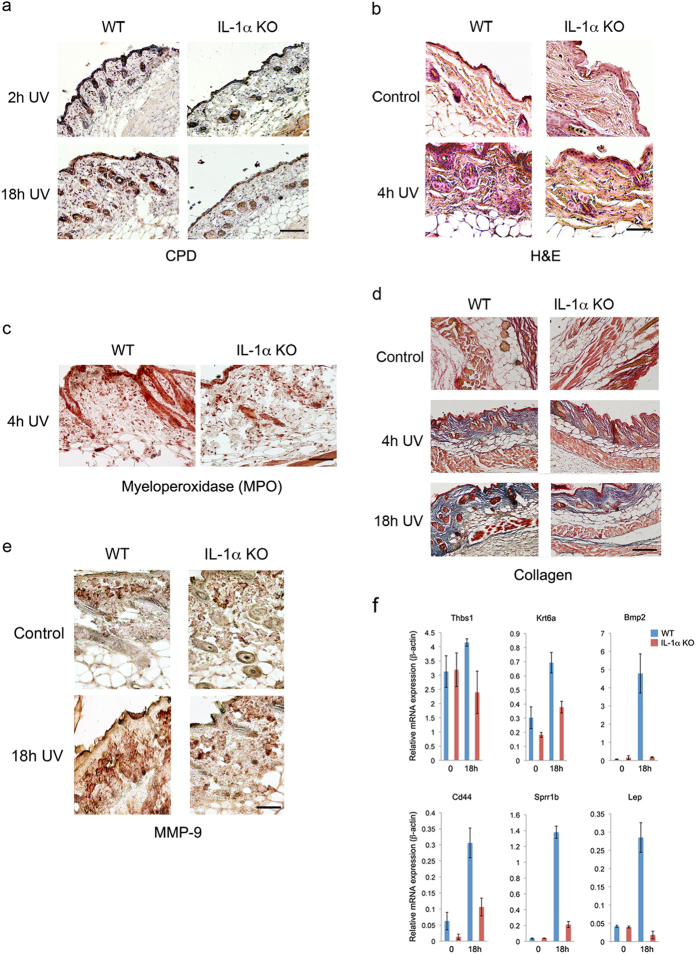
IL-1α is implicated in DNA damage associated skin inflammation, efficient wound healing and tissue repair. (**a**) IL-1α deficiency does not have a detectable effect on UV-induced epidermal Cyclobutane Pyrimidine Dimers (CPD) staining. WT and IL-1α KO mice were exposed to UV and sacrificed at different time points (2 h, and 18 h, n = 5 for each time point). Skin samples were obtained and analyzed for CPDs using immunohistochemistry. Staining at 2 h after UV exposure was used as control. While no obvious differences could be observed in CPD removal after 18 h, remarkable skin swelling is observed in WT but not in IL-1α KO mice skin samples. (**b**) IL-1α deficiency results in impaired skin inflammation and leukocyte infiltration after UV exposure. Paraffin-embedded skin samples from non-exposed (control) or UV exposed (4 h UV) WT and IL-1α KO mice were subjected to Hematoxylin and Eosin (H & E) staining. Representative snapshots of micrographs of H & E staining are shown. (**c**) IL-1α is important for neutrophil recruitment to the damaged site. Immunohistochemical staining of myeloperoxidase (MPO) in skins of WT or IL-1α KO mice 4 h after UV exposure (4 h UV. (**d**) WT and IL-1α KO mice were exposed to UV and were sacrificed at different time points (Control-non-exposed skin, 2 h, 4 h and 18 h hours after UVB exposure). Skin samples were treated and processed as described in Materials and Methods and stained with Masson’s trichrome to visualize Collagen fibers (blue) (**e**) Reduced MMP-9 induction in IL-1α deficient skins in response to UV exposure. Immunohistochemical staining of MMP-9 in WT or IL-1α KO mice skins before (control) or 18 h (18 h UV) after UV exposure. For quantifications see Supplementary Figure 4. (**f**) IL-1α is required for correct expression of wound healing related genes after skin DNA damage. RNA was extracted from the formalin-fixed, paraffin-embedded (FFPE) skin tissue sections and qPCR for the indicated genes was performed to monitor gene expression after cDNA synthesis. Data is expressed as mean ± SD of three independent samples.

To assess the potential physiological significance of IL-1α in DNA damage sensing, we performed immunohistochemistry (IHC) on shaved, UV irradiated skin from WT and IL-1α KO^25^. Despite the fact that CPD staining indicated no obvious differences in DNA damage between WT and IL-1α KO skin after 18 h (Fig. 4a), skin swelling and immune cell infiltration (Fig. 4b), which are the hallmarks of UV skin tissue damage^26^, were more prevalent in the skin of WT mice (n = 3). The fact that infiltration of cells was severely reduced in skin from IL-1α KO mice suggests that the “alarmin” activity of IL-1α could be important for UV damage-associated skin inflammation. In accordance with previous reports for IL-1α alarmin activity^5^,^18^,^27^, most of the UV associated inflammatory immune cells recruited by IL-1α were neutrophils as suggested by myeloperoxidase (MPO) staining (Fig. 4c and Supplementary Figure S4a).

Since recombinant IL-1α has been reported to enhance epidermal wound healing by stimulating fibroblast and keratinocyte growth and to induce collagen synthesis by fibroblasts in animal models^28^, we evaluated whether IL-1α deficiency may also affect tissue repair and wound healing *in vivo*. In parallel to decreased or delayed inflammation in skin from IL-1α KO mice, collagen formation, after its breakdown by UV (Fig. 4d, Supplementary and Figure S4b) and expression of Matrix Metallopeptidase 9 (MMP-9) (Fig. 4e and Supplementary Figure S4c) seem to be impaired 18 h post radiation in the skin of IL-1α KO mice, suggesting that IL-1α deficiency delays wound healing and tissue remodeling after UV induced skin damage. In addition, IL-1α seems to be essential for the expression of several wound healing related genes (e.g. Leptin (Lep), CD44, *Krt6b*, *Sprr1b*, *THBS1* and *Bmp2*)^29^, since their induction after UV exposure was decreased in IL-1α KO mice in comparison to WT mice (Fig. 4f). Several of these wound-healing genes are associated with TGF-β signaling, which is a key regulator of the wound healing response^30^,^31^.

Overall, our results suggest that the classical cytokine IL-1α can also act as an intracellular DNA damage sensor and report for cellular genotoxic stress. This novel mechanism can contribute to downstream tissue repair and wound healing processes and may potentially effect development of autoimmunity or cancer. The physiological significance of this newly discovered pathway and the role of IL-1α in efficient wound healing is supported by the observation that mice lacking either MMP-9^32^ or the adapter protein of the interleukin-1 receptor myeloid differentiation primary response gene 88 (MYD88)^33^ show impaired wound healing.

To date, the accepted immunologic danger model suggest that the immune system discriminates between dangerous and safe signals by recognition of pathogens or “alarm” signals from cells and tissues when cells are disrupted after injury, infection or oncogenic transformation^34^. Damage or danger associated molecular patterns (DAMPs) which are normally masked by cell compartmentalization are passively released by losing cell membrane integrity (e.g. traumatic cell death or necrosis) and activate the innate immune system. Our data adds a new layer to this concept of common danger theory by showing that “alarmins” could also sense and report cellular damage without the need for cell integrity breakdown.

Additionally, by identifying acetylation as a novel regulator of IL-1α cellular localization and secretion we uncovered a new role for HDAC’s in cytokine secretion, a mechanism that may also influence other cytokines. These findings would help to better understand the anti-inflammatory effects of HDAC inhibitors. Indeed HDAC inhibitors are now used to treat patients with severe acute graft-versus-host disease (GVHD)^35^ and systemic onset juvenile arthritis^36^ where levels of IFN-γ, TNF-α and IL-1α drop showing their potential as potent anti-inflammatory or immuno-modulatory effects at non-cytotoxic doses. Our data additionally suggest that blocking IL-1α or HDAC inhibition may be beneficial for reducing disease severity and symptoms^37^ in a broad spectrum of diseases with persistent DNA damage. Furthermore, uncontrolled IL-1 signaling may lead to acute pathogenesis, chronic diseases, autoimmunity and may promote tumorigenesis^12^,^18^,^19^.

## Methods

### Induction of high-resolution DNA damage

Single cells were irradiated with a femtosecond fiber laser coupled to a confocal microscope as described^38^. The pulse wavelength was 775 nm (pulse duration 250 fs, repetition rate 40 MHz). The peak power density at the focal plane was 660 GW/cm^2^ for analysis in fixed cells and 1050 GW/cm^2^ for recruitment studies in live cells. Cells were irradiated along a single track or two intersecting lines, each with a length of 6 μm. Recruitment kinetics were analyzed as described^39^.

### Immunofluorescence staining

6 × 10^4^ B16 mouse melanoma cells expressing mouse IL-1α-GFP were plated in IBIDI (2 ml) dishes and left to incubate overnight. Cells were either treated with Etoposide 10 μg/mL for 2 h (EBEWE Pharma) or laser irradiation as described above, followed by fixation with 1 ml 4% PFA for 20 min at room temperature (RT). For staining, all antibodies were incubated with cells overnight at 4 °C in a wet chamber: γH2AX (Millipore 5636), HDAC-1 (Active Motif 39531), HDAC-2 (Active Motif 39533), Anti-PML Protein antibody (ab53773) and CPD (Cosmobio, ordered from Hölzel Diagnostika, CAC-NM-DND-001) were used.

### Confocal microscopy and live cell imaging

Fluorescent images of mouse melanoma cells stably expressing GFP tagged IL-1α were collected using a Zeiss Cell Observer® spinning disc (SD). During the experiments, the cells were kept at 37 °C and 5% CO_2_, using a Tokai Hit incubation unit. GFP fluorescence was excited using a 488 nm diode laser and the emission was collected using a 500–550 nm BP (band pass). H_2_O_2_ treatment was performed at a concentration of 100 *μ*M, and images were collected every half hour for a period of 3 hours. TSA was used at a concentration of 100 ng/ml. Images were collected every hour for a period of 22 hours; representative images for three time points (0, 11, and 22 hrs. post treatment) were selected and presented in Fig. 3e. For each treatment and time point, regions of interest were selected in both the cytoplasm and nucleus for ~10 cells. Average fluorescence intensities were plotted using Origin 6.0 and are presented in Supplementary Figure 1b.

### Vectors and transfections

PrecIL-1α fused to GFP was cloned into the pEGFP-N1 vector (Clontech) as was previously described by Cohen *et al.*^5^. The substitute mimicry mutations of IL-1α-K82 was performed using the site-directed mutagenesis PCR reaction with the forward primer 5′-phospho-TCAAGCAACGGGCAGATTCTGAAGA or 5′-phospho-TCAAGCAACGGGCGGATTCTGAAGA together with reverse primer 5′-phospho-CGTTGCTGATACTGTCACCCGG with the precIL-1α-GFP encoding vector as a template and the Phusion DNA polymerase enzyme (New England Biolabs), according to the manufacturer’s protocol. For transient transfections, 5 × 10^4^ B16 mouse melanoma cells were cultured in 35 mm Fluorodish plates (World Precision Instruments) for 24 h and then transfected with the various IL-1α plasmids using the JetPEI transfection reagent (Polyplus Transfection). 16 h after transfection, the GFP signal was visualized with the Olympus Fluoview FV1000 confocal microscope (Olympus) using 405 nm and 488 nm laser lines. Nuclear counter-staining of the transfected cells was performed with Hoechst 33342 (Fluka).

### Cellular stress, ELISA, immunoprecipitation assays and FACS

1 × 10^5^ human HT-1080 or HaCAT cells were plated in triplicates in 6 well plates and allowed to grow overnight. Medium containing either 50 μgr/ml Bleomycin (SERVA) or 10 μM H_2_O_2_ was added to the plates for 16 h. To induce UV DNA damage, we used the same conditions as above but the medium was aspirated and the cells were irradiated with 5 mJ/cm^2^ and allowed to recover for an additional 16 h. For probing secreted IL-1α after genotoxic stress induction, the plates were centrifuged to eliminate floating cells and debris and the medium was collected and further analyzed by mouse or human IL-1α ELISA kits (all purchased from R&D Systems) and also for Lactate Dehydrogenase Activity Assay Kit (Sigma) or Cell Death Detection (ROCHE). In parallel, the same cell pellets were collected and apoptosis was assessed with TACS Annexin V-FITC Apoptosis Detection Kit (R&D) using Fluorescence Activated Cell Sorting (FACS).

IL-1α IP from cells of mouse Raw 264.7 macrophage line was performed with 1 × 10^8^ cells that were first induced with 100 ng/ml LPS (S. min.R595) for 12 h to enhance IL-1 expression. The cells were then washed 3 times with ice cold 1 × PBS, lysed with a glass tissue douncer in cell lysis buffer (PIERCE) and centrifuged for 20 min at 4 °C, 20,000 g to remove cell debris. 5 mg of total soluble protein from the supernatant was used for each IP. IL-1α proteins were precipitated with 5 μg of goat anti-mouse IL-1α (R&D Systems). Samples were then divided into 3 parts and equal loads were separated over 15% SDS PAGE and blotted with either PanAcetlysine (NEB) or monoclonal-hamster anti-mouse IL-1α (Santa Cruz clone ALF-161) or delivered for MS analysis. TSA inhibition of IL-1α secretion from Raw 264.7 cells was performed as described above for the cellular genotoxic assays. Medium containing 100 ng/ml TSA was added to the cells for 30 min and then removed and replaced with medium containing 100 ng/ml LPS. 12 h later, supernatants were collected and secreted IL-1α and IL-6 were measured with mouse ELISA kits (R&D Systems). For WT IL-1α and K82 mimicry mutation, B16 melanoma cells were plated in triplicate in 6 well plates and transiently transfected with the indicated plasmids. The cells were allowed to express GFP-IL-1α and left to recover for an additional 24 h. To induce DNA damage, UV was applied as described above and levels of secreted GFP-IL-1α were measured by measuring GFP using the PathScan total GFP Sandwich ELISA kit (Cell signaling). To normalize for transfection efficiencies between the different GFP tagged plasmids, we also measured total GFP signals for cell lysates after cell pellets were lysed using the cell lysis buffer provided with the PathScan. Relative (secreted/total cell) GFP concentrations are presented in Fig. 2d. To evaluate the expression of GFP-IL-1α and K82 mimicry mutations in transiently transfected B16 melanoma cells, total proteins from 3.5 × 10^5^ GFP positive FACS sorted cells expressing the different constructs were separated over 15% SDS-PAGE and GFP signals were probed with anti GFP (Ab290).

### Total cell protein extraction

Protein extracts were prepared from 1 × 10^6^ human HT-1080 or HaCAT cells. After cell trypsinization, pellets were washed 3 times with ice-cold 1 × PBS and lysed with 100 μl of RIPA lysis buffer (50 mM Tris, 150 mM NaCl, 2 mM EDTA 1% NP-40, 0.1% SDS and 1 mM PMSF pH = 7.5) for 10 min at 4 °C. Supernatants were cleared of debris by 10 min centrifugation at 13,000-rpm at 4 °C and total protein concentrations were determined using the Roti-Nanoquant assay (Carl Roth). Equal protein loads were supplemented with 5× SDS laemmli loading buffer and boiled for 10 min at 95 °C. Approximately 5 μg of total cell lysates were separated over 15% SDS-PAGE, transferred to a 0.45 μm nitrocellulose membrane (Whatman) and blotted with mouse monoclonal anti-human IL-1α (sc-73494 Santa Cruz Biotechnology).

### UV exposure and immunohistochemistry staining

C57BL/6 WT mice were purchased from Harlan Laboratories (Rehovot, Israel). IL-1α KO mice were generated in the laboratory of Prof. Y. Iwakura^25^. All animals were bred and kept at the Animal Facilities of the Faculty of Health Sciences, Ben-Gurion University, Beer-Sheva, Israel. The Animal Care Committee of Ben-Gurion University approved all animal studies in this research. For UV exposure, mouse backs were first shaved and then exposed to UV radiation (5000 J/m^2^) using a VL-6 UV lamp (VILBERLOURMAT, France). 2, 4 and 18 hours after UV exposure, a section of the exposed skin was removed and fixed in 4% paraformaldehyde, dehydrated in alcohol, cleared with xylene and embedded in paraffin. Four-micron sections were stained with H&E, using established protocols. For collagen staining, the Masson trichromica kit (Bio-Optica, Milano, Italy) was used, following the standard protocol. Immunohistochemistry of irradiated skin tissue sections was performed as described previously and after appropriate retrievement and blocking, slides were stained with the indicated primary antibodies: anti-CPD (Cosmobio, CAC-NM-DND-001); rabbit polyclonal anti-myeloperoxidase (MPO) (Abcam, Cambridge, UK) and monoclonal anti-MMP9 (Abcam, Cambridge, UK). The Vectastain Elite ABC Peroxidase kit or the Universal ImmPRESS kit (Vector Laboratories, Inc, Burlingame, CA) was used as the secondary antibody and visualization was performed using AEC as a substrate (ZYMED Laboratories Inc, San Francisco, CA).

### Mass Spectrometry: MS/MS identification of acetylated IL-1α

*In vivo* modified IL-1α was enriched via IP with hamster monoclonal anti-mouse IL-1α antibody (ALF-161, Santa Cruz Biotechnology) from mouse leukemic monocyte macrophages in the presence of 5 mM sodium butyrate (Sigma) and separated on a bis-tris gel with MOPS running buffer (NuPAGE, Invitrogen). The silver stained band corresponding to the precIL-1α was digested with trypsin (Promega) and analyzed by nanoLC-MS essentially as described^40^ with minor changes.

Briefly, STAGE tip desalted samples were analyzed using nano-flow (Agilent 1200 nanoLC, Germany) LC-MS/MS on a linear ion trap (LIT)-Orbitrap (LTQ-Orbitrap XL) mass spectrometer (ThermoFisher, Germany). Peptides were eluted with a linear gradient of 10–60% buffer B (80% ACN and 0.5% acetic acid) at a flow rate of 250 nL/min over 60 min. MS data were processed into peak lists by DTASuperCharge 2.0b1 (part of the MSQuant 2.0b7 software environment) and searched with Mascot 2.2 against the mouse International Protein Index protein database (IPI, version 3.68) combined with frequently observed contaminants and concatenated with the reversed versions of all sequences. The MMD for monoisotopic precursor ions and MS/MS peaks were restricted to 5 ppm and 0.6 Da, respectively. The enzyme specificity was set to trypsin (with a maximum of 2 missed cleavages) allowing cleavage of the N-terminal to proline and C-terminal to aspartate. Modifications were cysteine carbamidomethylation (fixed), as well as protein lysine acetylation, serine/threonine/tyrosine phosphorylation, deamidation (asparagine and glutamine) and methionine oxidation (variable). Protein and peptide identifications were further analyzed and manually annotated by inspection of chromatograms and spectra (see Fig. 3B).

### RNA extraction from formalin-fixed paraffin-embedded (FFPE) skin tissue sections and real-time PCR

Formalin-fixed paraffin-embedded (FFPE) skin tissue sections were deparaffinized with 2× histosol baths to remove paraffin followed by 2× ethanol, 100 °C washes which are miscible with histosol and 2 ethanol 95 °C baths (3 minutes for each bath). For rehydration, tissues were incubated in a distilled water bath for 2 h before RNA extraction was preformed. To achieve a high RNA yield per sample, 2 sections from each skin sample and 2 time points were combined from a total number of 3 mice from each mouse type (e.g WT or IL-1α KO and time 0 or 18 h after UV exposure). Total RNA was extracted using the RNeasy FFPT kit (QIAGEN) and 500 ng of total RNA was used for cDNA synthesis using the RevertAid H Minus First Strand cDNA Synthesis Kit (Thermo Scientific). qPCR reactions were all preformed with LightCycler 480 II real-time PCR (Roche). All qPCR primers were previously described in Kim *et al.* 2013^29^. Data were normalized to the expression of the housekeeping gene β-actin.

### Digital Image Analysis

Digital images were prepared for analyses using the ImageJ. In each image, the area of interest (collagen-blue) was selected and separated from the rest of the field using the Color Threshold tool and analyzed according to hue, saturation and brightness settings. The number of stained pixels was then divided by the number of pixels in the rest of the tissue, in order to calculate the percentage of collagen-specific staining. 4 randomly chosen fields within of 4 independent IHC stains (×400) (4 mice per group) were used from 3 independent experiments.

## Additional Information

**How to cite this article**: Idan, C. *et al.* IL-1α is a DNA damage sensor linking genotoxic stress signaling to sterile inflammation and innate immunity. *Sci. Rep.* **5**, 14756; doi: 10.1038/srep14756 (2015).

## Supplementary Material

Supplementary Information

Supplementary Video 1

Supplementary Video 2

Supplementary Video 3
