# Corrigendum: IL-1α is a DNA damage sensor linking genotoxic stress signaling to sterile inflammation and innate immunity

**DOI:** 10.1038/srep19100

**Published:** 2016-01-11

**Authors:** Idan Cohen, Peleg Rider, Elena Vornov, Martin Tomas, Cicerone Tudor, Mareike Wegner, Lydia Brondani, Marina Freudenberg, Gerhard Mittler, Elisa Ferrando-May, Charles A. Dinarello, Ron N. Apte, Robert Schneider

Scientific Reports 5: Article number: 1475610.1038/srep14756; published online: 10062015; updated: 01112016

The original version of this Article contained errors in the spelling of the authors Idan Cohen, Peleg Rider, Elena Vornov, Martin Tomas, Cicerone Tudor, Mareike Wegner, Lydia Brondani, Marina Freudenberg, Gerhard Mittler, Elisa Ferrando-May, Ron N. Apte and Robert Schneider which were incorrectly given as Cohen Idan, Rider Peleg, Voronov Elena, Tomas Martin, Tudor Cicerone, Wegner Mareike, Brondani Lydia, Freudenberg Marina, Mittler Gerhard, Ferrando-May Elisa, Apte N. Ron and Schneider Robert respectively. These errors have now been corrected in the PDF and HTML versions of the Article.

