# Supplementary material for: IL-1α is a DNA damage sensor linking genotoxic stress signaling to sterile inflammation and innate immunity
Source: Sci Rep. 2015 Oct 6;5:14756. doi: 10.1038/srep14756 (PMC4593953; doi:10.1038/srep14756)

IL-1α is a DNA damage sensor linking genotoxic stress signaling to sterile inflammation and innate immunity

Running title: IL-1α linking genotoxic stress to sterile inflammation

Cohen Idan[1, 2]*, Rider Peleg[3], Voronov Elena[3], Tomas Martin[4], Tudor Cicerone[2], Wegner Mareike[2,5], Freudenberg Marina[2,7], Mittler Gerhard[2], Ferrando-May Elisa[4], Charles A. Dinarello[8], Apte N. Ron[3*] and Schneider Robert1[1]*.

Supplementary information

Supplementary figures

Supplementary Figure 1 (a) Precursor IL-1α is expressed in non-treated human HT1080 fibrosarcoma and HaCaT human keratinocyte. Total cell lysates were separated over 15% SDS PAGE and hIL-1α was detected using mouse anti-hIL-1α monoclonal antibody. (**b**) The IL-1α signal increases in the cytoplasm after DNA damage induction. Live cell imaging of B16 melanoma cells expressing GFP-IL-1α after 100 μ M H_2O_2 treatment. Images were collected in intervals for a total period of 4 hours. For every condition (treatment and non treated) a representative region was selected in both the cytoplasm and nucleus for quantification. Averaged fluorescence intensities (~10 cells) of cytoplasmic regions (left) or nuclear/cytoplasm ratio (right) are plotted as mean ± SD of 10 single cell measurements. (**c**) Quantification of IL-1α co-localization with γH2AX within DNA damage induced foci. For measuring florescence intensities, automatic foci detection using IMARIS software was applied to measure and evaluate florescence intensities and co-localization. The quantification of co-localization is expressed as the percentage of positive γH2AX foci co-localizes with high IL-1α intensity (e.g. above background of non foci intensities).

Supplementary Figure 2 (**a**) B16 melanoma cells expressing GFP-IL-1α were irradiated with high UV dose (250 $mJ/cm^{2)}$ to induce apoptosis (upper panel). 2-3h post irradiation, GFP-IL-1α immobile apoptotic foci were visualized by confocal microscopy. For compression, low UV dose of (5 mJ/cm^2) was used to show the different foci formation 2h post irradiation upon genotoxic stress (low panel). γH2AX staining was used to visualize DNA damage foci (red). (**b**) B16 melanoma cells expressing GFP-IL-1α were UV irradiated at 5 mJ/cm^2 to induce DNA damage. IF using PML specific antibodies was carried out to determine co-localization with IL-1α under genotoxic stress. White bars, 20 μ m. (**c**) Detection of apoptoic cell death after genotoxic stress. HT1080 fibrosarcoma or HaCaT keratinocytes were exposed to several genotoxic stresses as in figure 2 (e.g. UV H_2O_2 and bleomycin). Cell pellets were collected and analyzed for Annexin V-positive staining by Fluorescence Activated Cell Sorting (FACS). Sub-lethal dose of H_2O_2 (250μM) (high H_2O_2) serve as positive control. Data is expressed as mean ± SD.

Supplementary Figure 3 (a) Precursor IL-1α K82 point mutants do not effect its transient ectopic expression in B16 mouse melanoma cells. B16 melanoma cells were transfected with the different precIL-1α GFP vectors and FACS sorted for GFP positive signal. Total protein extracts from $3.5X10^5$ cells were separated over 15% SDS PAGE and

precIL-1α GFP levels were probed with anti GFP rabbit polyclonal antibody. Histones serve as loading control. **(b)** TSA treatment increases nuclear/cytoplasmic IL-1α ratio. B16 melanoma expressing GFP-IL-1α were either untreated (control) or treated with TSA 100ng/ml. Images were collected every 30 min for a period of 22h. For every condition, regions of interest were selected in both the cytoplasm and nucleus for ~10 cells. Averaged fluorescence ratios between nuclear and cytoplasmic signal were plotted using Origin 6.0. **(c)** TSA treatment decreases IL-1α and IL-6 secretion from Raw 264.7 mouse macrophages. TSA (100ng/ml) was added to the cells for 30 min. Growth medium was then replaced with medium containing 100ng/ml LPS. 12h later secreted IL-1α and IL-6 were measured in supernatants using specific ELISA kits. The experiment was performed in triplicate and data is expressed as mean ± SD of the three independent experiments.

Supplementary Figure 4 Numbers of MMP-9-positive cells (**a**) collagen intensity (**b**) and MPO-positive cells (**c**) in WT or IL-1α KO IHC. IHC stains were analyzed by ImageJ or manually counted in stains or sections from 4 randomly chosen fields out of 4 independent IHC stains (x400) (4 mice per group) from 3 independent experiments. Results are presented as mean of positive cells or collagen intensity ± SD. ns: non-significant, *P<0.05, **P<0.01 and ***P<0.001.

Supplementary video 1. Nuclear/cytoplasmic shuttling of IL-1α after DNA damage sensing. Live cell imaging of melanoma cells expressing GFP-IL-1α during treatment with 100 μM H_2O. First 5s show un-induced cells.

Supplementary video 2 and 3 IL-1α localizes to DNA damage sites. Single B16 melanoma cells expressing IL-1α–GFP were laser microirradiated along a single track for induction of DNA damage. IL-1α localisation to the site of damage can be seen by increased GFP intensity along intersecting lines. Video 2 and 3 are two independent replicate experiments.

Cohen et al. supplementary fig 1

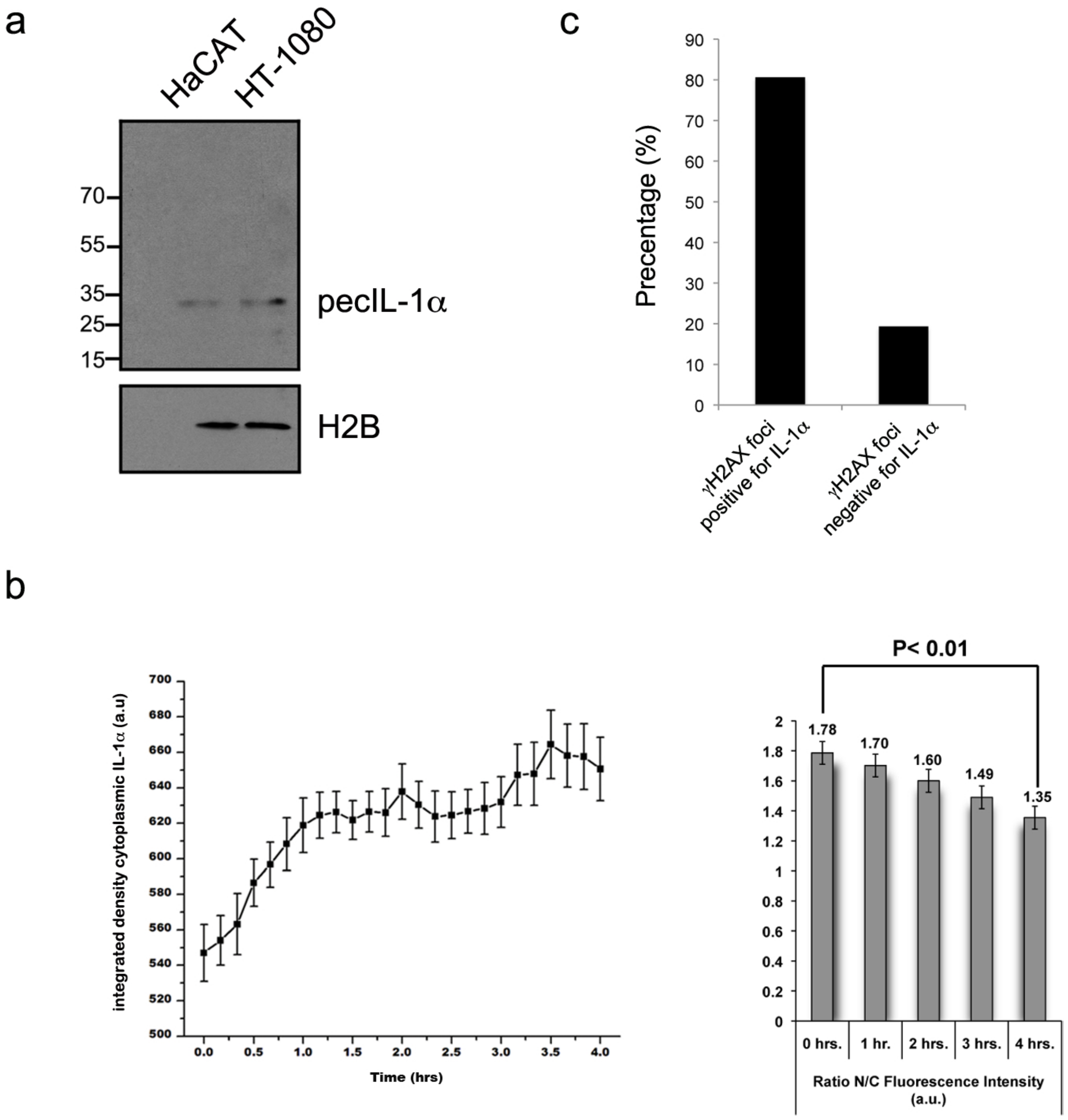

Cohen et al. supplementary fig 2

a

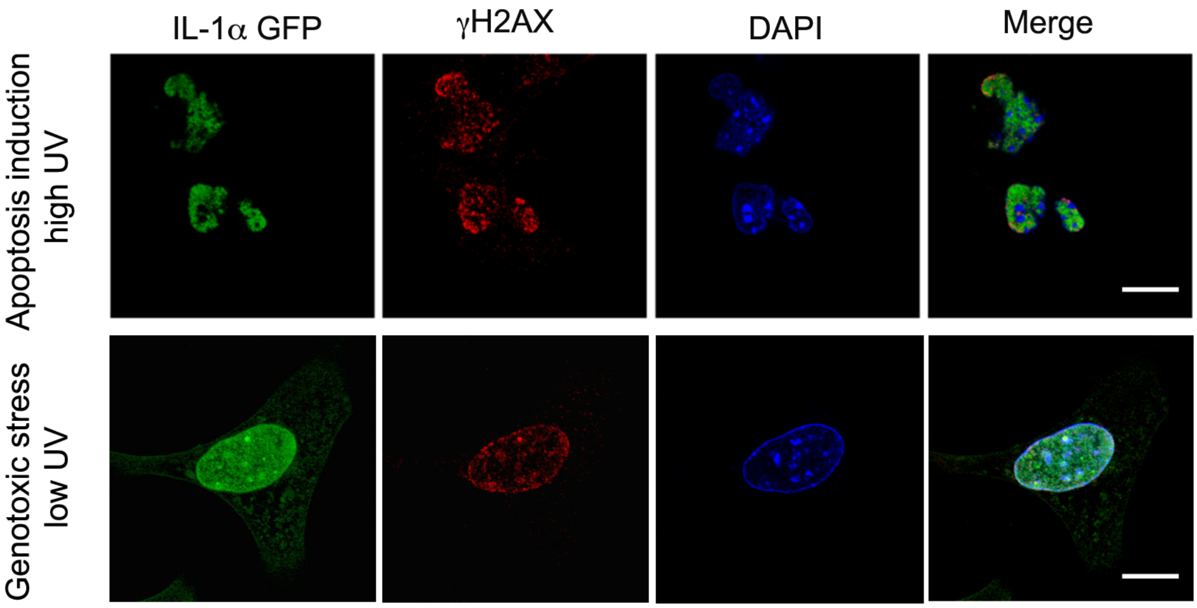

b

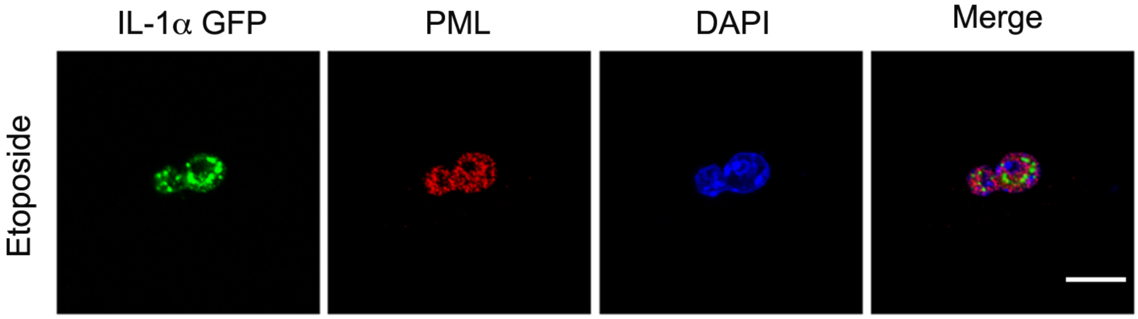

c

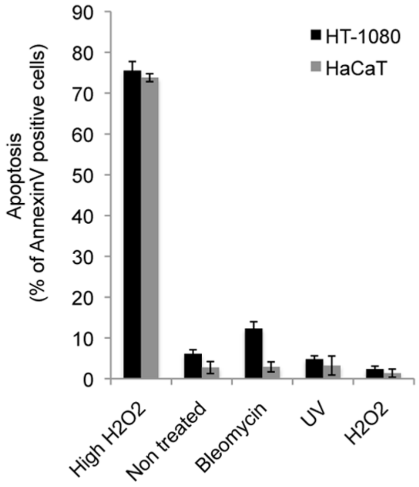

Cohen et al. supplementary fig 3

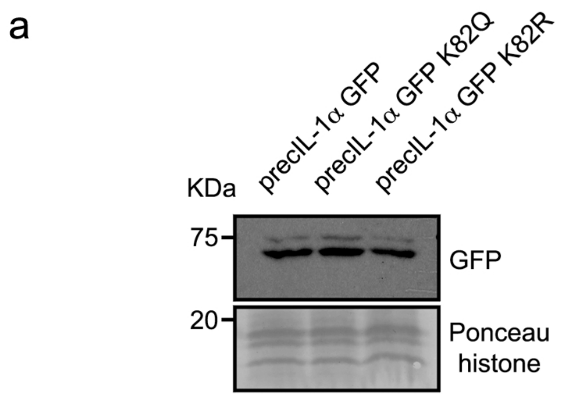

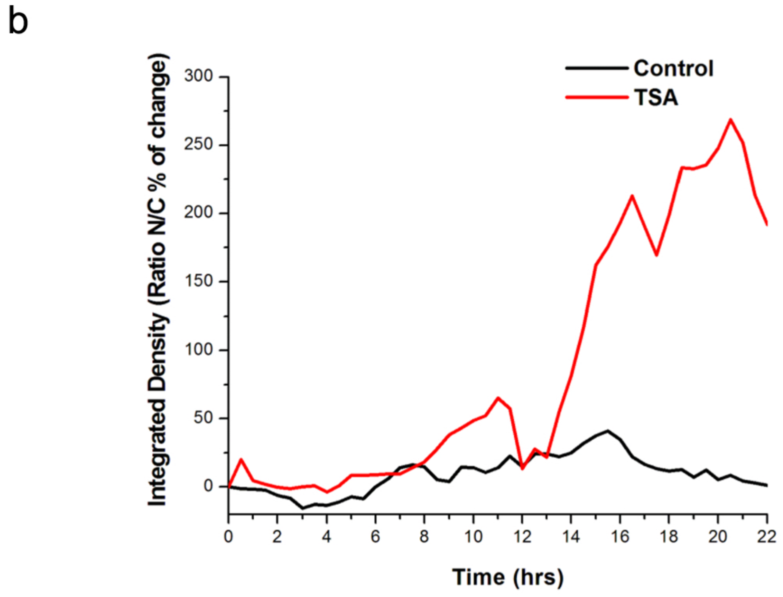

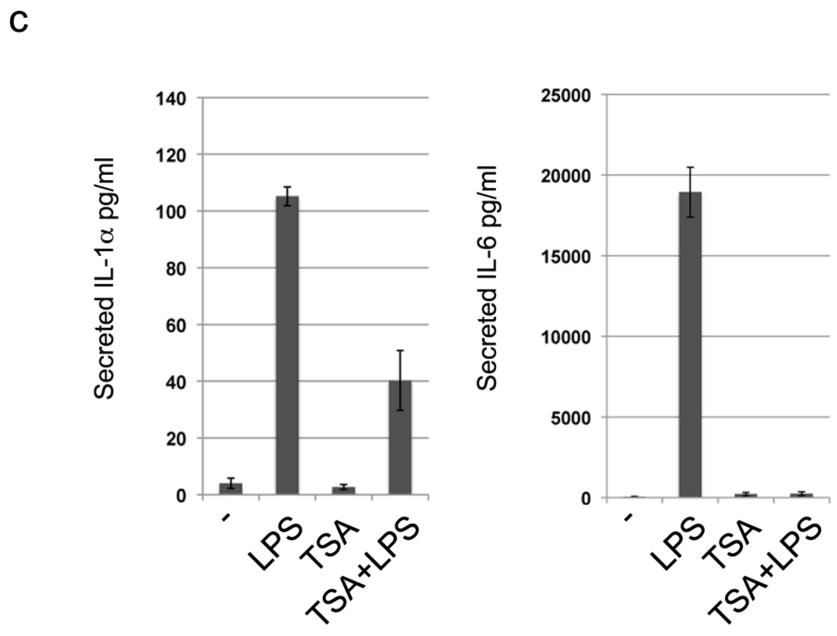

Cohen et al. supplementary fig 4

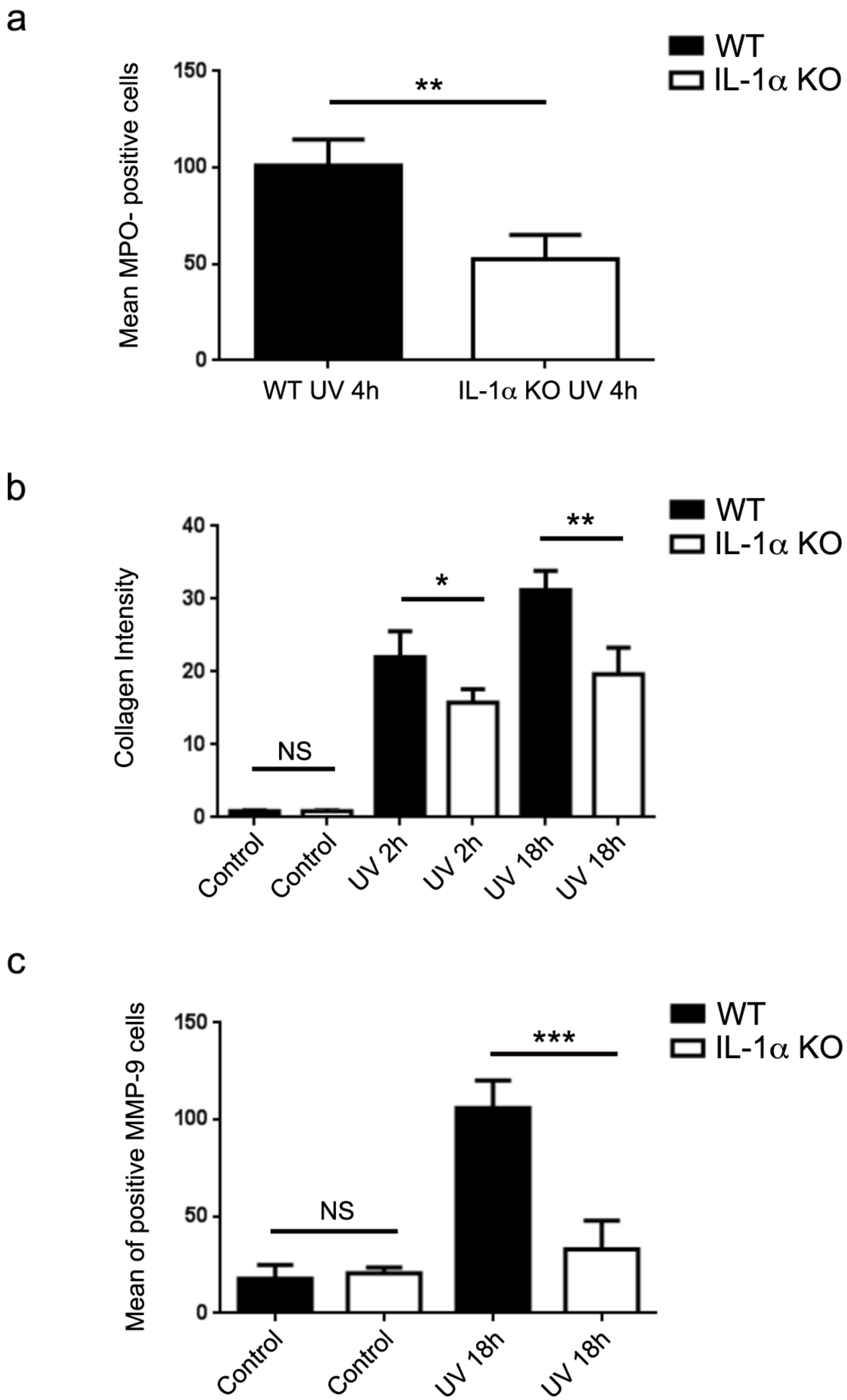

Supplement: Supplementary Information [file srep14756-s1.pdf]
